# Celastrol reduces cisplatin-induced nephrotoxicity by downregulating SNORD3A level in kidney organoids derived from human iPSCs

**DOI:** 10.3389/fphar.2025.1464525

**Published:** 2025-03-27

**Authors:** Chongayng Shen, Qizheng Wang, Xun Ye, Yi Zhou, Huayang Xing, Chengjie Pan, Heying Li, Chunjie Wu, Mingliang You

**Affiliations:** ^1^ Basic Medicine School, Chengdu University of Traditional Chinese Medicine, Chengdu, China; ^2^ CAS Key Laboratory of Regenerative Biology, Joint School of Life Sciences, Guangzhou Institutes of Biomedicine and Health, Chinese Academy of Sciences, Guangzhou Medical University, Guangzhou, China; ^3^ School of Pharmacy, Chengdu University of Traditional Chinese Medicine, Chengdu, China; ^4^ Hangzhou Aimingmed Organoids Bank, Hangzhou, China; ^5^ The Fourth Clinical Medical College of Guangzhou University of Chinese Medicine, Guangzhou University of Chinese Medicine, Shenzhen, China

**Keywords:** celastrol, kidney organoids, nephrotoxicity, cisplatin, SNORD3A

## Abstract

**Background:**

Celastrol, an active ingredient derived from *Tripterygium wilfordii Hook F*, has shown therapeutic potential for various kidney renal diseases. The kidney protective activity of celastrol is mainly exerted through anti-inflammatory, and antioxidant effects. However, celastrol causes dose-dependent kidney toxicity, which results in increased risks of mortality among patients. This study aimed to develop a kidney organoid-based prediction system to assess the safety and efficacy of celastrol in reducing cisplatin-induced nephrotoxicity.

**Methods:**

We investigated the ability of celastrol to reduce cisplatin-induced nephrotoxicity using kidney organoids. Kidney organoids were cultured and characterized, exhibiting renal tubular and glomerular structures and expressing specific kidney markers such as NPHS1, CD31, LTL, and SLC12A1. Data were obtained from *in vitro* experiments in which kidney organoids were exposed to therapeutically relevant concentrations or a toxic dosing profile of cisplatin and celastrol, to assess their impact on cell viability using flow cytometry and Acridine Orange/Propidium Iodide (AO/PI) staining. In addition, RNA-seq analyses were performed to determine the mechanisms of celastrol function in the kidney.

**Results:**

Kidney organoids exposed to 50 µM cisplatin showed significantly increased cell death (only 0.37% cells with normal cell structure), whereas celastrol under 5 µM (56% cells with normal cell structure) showed significantly less nephrotoxicity than cisplatin. The protective effects of celastrol against cisplatin-induced nephrotoxicity were further investigated by treating the organoids with both compounds. The results demonstrated that 2 µM celastrol reduced cisplatin-induced nephrotoxicity by downregulating SNORD3A and HIST1H3A gene levels.

**Conclusion:**

This study highlights the potential of celastrol as a protective compound against cisplatin-induced kidney damage and emphasizes the importance of using advanced models, such as iPSC-derived kidney organoids, to predict therapeutic effect and nephrotoxic concentrations of novel drugs.

## Introduction

Chinese medicine has been used clinically to treat various diseases for thousands of years. Its acceptance has grown in recent years because of its therapeutic effectiveness (Xin Luan et al., 2020). As society has progressed and developed, it has gained popularity around the world and is now generally regarded as a supplement and an alternative therapy in many countries (Zhang et al., 2021). According to available data, the traditional Chinese medicine business is enormous, with Chinese herbs exported to more than 175 countries and territories, including Japan, South Korea, India, Germany, the Netherlands, European countries, and the United States (Yang et al., 2018).

Many Chinese herbal medicines have good therapeutic effects but can cause adverse effects such as nephrotoxicity (Shi et al., 2020). For example, several contain nephrotoxic components, such as aristolochic acids and other alkaloids, that can lead to kidney problems such as acute kidney injury and chronic kidney disease. Celastrol, derived from *Tripterygium wilfordii* Hook F, has anti-inflammatory and antitumor properties (Boran et al., 2019), treats renal diseases, and has nephrotoxic effects (Wu et al., 2021). Certain concentrations of celastrol have been found to be toxic, whereas lower doses have demonstrated a protective effect against kidney damage. Understanding the dual effects and dose-dependent toxicity of celastrol is essential for its safe and effective use in clinical settings. According to Jiang et al. (Jiang et al., 2013), high concentrations of celastrol (>1.0 μM) caused G2/M arrest and apoptosis in Huh7 cells by activating caspase3/7, whereas low concentrations (<1.0 μM) exhibited no evident effects. Low-concentration celastrol had substantial combinatorial effects with Phytohemagglutinin (PHA) on Huh7 cells and Huh7 xenografts, inhibiting proliferation and migration and inducing apoptosis. Zhang et al. (Jianhe) discovered that large doses of it worsened renal damage in model rats. Although it causes dose-dependent renal toxicity in normal rats, it also exerts a protective effect on the pathology of kidney damage at certain doses. It is vital to correctly comprehend the “two-way effect” of celastrol’s protection and damage, as well as its “dose-effect (toxicity) relationship”. Thus, greater attention should be directed toward the judicious use of celastrol and related preparations (Lianqi).

Nephrotoxicity, the second most common type of drug-induced damage in critically ill patients, accounts for approximately 25% of kidney failures in this group (Astashkina et al., 2012). Traditional animal models, which often use rats, mice, and rabbits, have limitations in accurately predicting human responses owing to interspecies differences and high costs. *In vitro* models utilizing proximal renal tubular cells, such as human and canine epithelial cell lines (ZHUANG Yan-shuang et al.), are commonly used for early nephrotoxicity screening. However, these models lack a three-dimensional tissue structure and inter-organ interactions, failing to fully represent the effects of drugs on kidney tissue. Human-derived *in vitro* models, particularly 3D kidney organoids from induced pluripotent stem cells (iPSCs), have been used (Wu et al., 2023). These organoids mimic the physical and functional aspects of human kidney tissues and include multiple cell types (Matsui and Shinozawa, 2021; Chen et al., 2021). Unlike 2D cultures, organoids can sustain specific cell phenotypes for extended periods, making them more suitable for high-throughput drug screening (Khoshdel Rad et al., 2020; Kim et al., 2020). Ueno et al. (2022) showed that kidney organoids are effective for nephrotoxicity assessment, offering comprehensive insights into the underlying mechanisms. Thus, iPSC-derived kidney organoids are a valuable platform for modelling organogenesis and evaluating human nephrotoxicity (Nauryzgaliyeva et al., 2023; Freedman et al., 2015).

Platinum-based chemotherapeutics, such as cisplatin, have been pivotal in cancer treatment for decades, particularly against lung, ovarian, brain, and breast cancers. Despite its effectiveness, its side effects (mainly nephrotoxicity) limit its clinical use (Shi et al., 2022). Several studies have found that celastrol can ameliorate cisplatin-induced nephrotoxicity via oxidative stress (Yu et al., 2018). This study focuses on develop the *in vitro* predictive assays using iPSC-derived kidney organoids, which more closely resemble the structure of adult kidney tissue, could help predict the potential protective effects of celastrol treatment against cisplatin-induced nephrotoxicity more accurately in humans.

## Materials and methods

### Human iPSCs culture

Human iPSCs were obtained from CAS key laboratory of regenerative biology. The cells were cultured on growth factor-reduced Matrigel (354277, Corning Life Sciences, Kennebunk, ME, United States)-coated 6-well plates (0.013 mg/cm^2^) in mTeSR medium (85875, Stemcell Technologies, Vancouver, BC, Canada) and cells were subcultured every 3–4 days at a ratio of 1:6.

### Self-organization of kidney organoids

The culture of iPSC-derived kidney organoids in this study was initiated according to the method proposed by Przepiorski et al. (2018). Before differentiation, iPSCs were maintained on a 10-cm Matrigel-coated cell culture dish to approximately 75% confluence. On day 0, embryoid bodies were generated by detaching colonies into single cells with1 mg/mL dispase (17105041, GIBCO, Grand Island, NY, United States) and then cells were collected at 200 g for 5 min by centrifugation at room temperature. The cells were resuspended in BPEL (supplemented with 8 mM CHIR99021 (S2924, Selleck Chemicals, Houston, TX, United States), 3.3 mM Y27632 (S1049, Selleck), and 1 mM β-mercaptoethanol (M3148, Sigma-Aldrich, St. Louis, MO, United States) and seeded into a 6-well ultra-low attachment plate (3,471, Corning). After 48 h, half of the medium was replaced with BPEL and CHIR99021 (8 mM). From day 3 onwards, embryoid bodies were resuspended in Stage II culture medium until day 14 and agitated daily to prevent excessive fusion. BPEL and Stage II medium (10–100–455, Aimingmed, Hangzhou, Zhejiang, China) were prepared as described by Przepiorski et al. (2018).

### Cisplatin and celastrol treatment

Cisplatin (p4394, Sigma) and celastrol (C0869-10 MG, Sigma) were used for the nephrotoxicity testing. Cisplatin was reconstituted with a 0.9% sodium chloride solution to achieve a stock concentration of 0.5 mg/mL, and celastrol was dissolved in DMSO to reach a stock concentration of 10 mg/mL. The organoids were exposed to 50 μM cisplatin, as well as 100 nM, 200 nM, 500 nM, 1 μM, 2 μM, 5 μM, 10 μM, or 50 μM celastrol for a duration of 2 days starting from day 11 *in vitro*. In order to assess the kidney protective effect of celastrol, either 1 μM or 2 μM celastrol was co-administered with the cisplatin at a concentration of 50 μM. Organoids were harvested for immunostaining after fixation with 4% paraformaldehyde (P0099, Beyotime, Shanghai, China), or for qPCR and RNA-seq using TRIzol (15596018, Invitrogen, Carlsbad, CA, United States).

### RNA extraction, cDNA synthesis and quantitative real-time PCR

Total RNAs was extracted using TRIzol, and 1 μg of total RNA was used for cDNA synthesis using the GoScript reverse transcription mix (A2790, Promega, Madison, WI, United States). Real-time PCR was performed using the SsoAdvanced SYBR Green Supermix (1725274, Bio-Rad, Hercules, CA, United States) in an real-time PCR machine (StepOnePlus, Thermo Fisher Scientific, Waltham, MA, United States). For the miRNAs, 2.5 μg of total RNA was reverse transcribed using All-in-One MiRNA Q-PCR Detection Kit (GeneCopoeia, Rockville, MD, United States). The primers used for the reaction are listed in Table 1.

**TABLE 1:** The primers used for RT-qPCR.

Gene	Forward primer (5′-3′)	Reverse primer (5′-3′)	
GAPDH	GGC​ATG​GAC​TGT​GGT​CAT​GAG	TGC​ACC​ACC​AAC​TGC​TTA​GC	
IL8	ACT​GAG​AGT​GAT​TGA​GAG​TGG​AC	AAC​CCT​CTG​CAC​CCA​GTT​TTC	
KIM1	TGT​CTG​GAC​CAA​TGG​AAC​CC	GGC​AAC​AAT​ATA​CGC​CAC​TGT	
MCP1	CAG​CCA​GAT​GCA​ATC​AAT​GCC	TGG​AAT​CCT​GAA​CCC​ACT​TCT	
IL1B	TTC​GAC​ACA​TGG​GAT​AAC​GAG​G	TTT​TTG​CTG​TGA​GTC​CCG​GAG	
HIST1H3A	CTA​GTG​TTG​GGT​GTT​CCG​CT	CTG​CCT​TAG​TGG​CCA​ACT​GT	
SNORD3A	CGG​TGA​CGG​CTC​TTG​GGT​TT	CGGGAAACGGCGACAAAA	
miR-3615	CTCGGCTCCTCGCGGCTC	GCAGGGTCCGAGGTATTC	
RPPH1	-GAG​CTG​AGT​GCG​TCC​TGT​C	TCA​GGG​AGA​GCC​CTG​TTA​GG	
U6	ATT​GGA​ACG​ATA​CAG​AGA​AGA​TT	GGA​ACG​CTT​CAC​GAA​TTT​G	

### RNA sequencing (RNA-seq) and bioinformatics analysis

To characterize the differential expression of RNA transcripts between the control, cisplatin-induced nephrotoxicity, and celastrol kidney protection groups, whole genome transcript sequencing was performed by (Aimingmed). Up-sequencing using the library construction method with rRNA removal allowed for simultaneous detection of mRNA expression levels. Gene expression was analyzed to assess the correlation between gene expression characteristics and differentially expressed genes within and between groups. Then, using pheatmap package and the hierarchical clustering was performed. The results were visualized using a heatmap. Kyoto Encyclopedia of Genes and Genomes (KEGG) pathway enrichment and Gene Ontology (GO) enrichment analyses were performed.

### Immunofluorescence analysis

The organoids were washed with PBS and fixed with 4% paraformaldehyde (PFA) for 1 h at room temperature (RT). Afterwards, they were incubated in sucrose (30% w/v) in PBS overnight at 4°C. Then, the organoids were embedded in optimal cutting temperature (OCT) compound (4,583, Tissue-Tek, Torrance, CA, United States) and cryosectioned into 10-µm sections. The primary antibodies and dilutions used for immunofluorescence analysis are listed in Table 2. After washing thrice with PBS, the sections were incubated with the corresponding secondary antibodies (Table 2) and diluted in PBS for 1 h at RT. The nuclei were counterstained with DAPI for 10 min. Images were obtained using a confocal microscope (LSM710, Zeiss, Oberkochen, Germany).

**TABLE 2:** The primary and secondary antibodies used for immunofluorescence analysis.

Antibody	Host species	Producer	Product code	Dilution
LTL	-	Vector Labs	FL-1321	1:300
KIM1	Goat	R&D Systems	AF1750	1:50
γH2AX	Rabbit	Cell Signaling	2577S	1:100
NPHS1	Sheep	R&D systems	AF4269-SP	1:200
CD31	Mouse	BD Biosciences	555444	1:200
SLC12A1	Rabbit	Sigma	HPA018107	1:200
PODXL	Mouse	R&D Systems	MAB1658	1:200
HNF1B	Rabbit	Sigma	HPA002083-100UL	1:200
CDH1	Mouse	BD Biosciences	610181	1:200
MEIS1/2/3	Mouse	Active Motif	39796	1:300
Donkey anti-Rabbit IgG (H + L) Cross-Adsorbed Secondary Antibody, Alexa Fluor™ 568	Donkey	Invitrogen	A10042	1:1000
Donkey anti-Sheep IgG (H + L) Cross-Adsorbed Secondary Antibody, Alexa Fluor™ 488	Donkey	Invitrogen	A-11015	1:1000
Donkey anti- Goat IgG (H + L) Cross-Adsorbed Secondary Antibody, Alexa Fluor™ 647	Donkey	Invitrogen	A-21447	1:1000
Goat anti-Mouse IgG (H + L) Cross-Adsorbed Secondary Antibody, Alexa Fluor™ 568	Goat	Invitrogen	A-11004	1:1000

### Statistical analysis

We used the GraphPad Prism (Version 7; GraphPad Software La Jolla, CA, United States) to conduct statistical analysis. Data were expressed as the mean ± SD. For comparison of more than three groups, Shapiro-Wilk test was used to check if a continuous variable follows a normal distribution. If P > 0.05, one-way ANOVA was applied; If p < 0.05, Kriskall-Wallis test was applied. Results were considered statistically significant with p values: ***p < 0.001, **p < 0.01.

### Flow cytometry

The organoids were washed with cold PBS and dissociated into single cells using Accumax (07921, STEMCELL). The cells were stained with propidium iodide (PI; Invitrogen) in PBS for 15 min at 4°C. The cells were washed with PBS and strained through a 100 μm mesh. Flow cytometry measurements were performed using a BD FACSCalesta cytometer (BD Diagnostics, Franklin Lakes, NJ, United States).

### Transmission electron microscopy

The organoids were collected in a 2 mL tube and fixed with an electron microscopy fixation buffer consisting of glutaraldehyde (2.5%), paraformaldehyde (2%), and phosphate buffer (PB, 0.1 M, pH 7.4) overnight at 4°C. Post-fixation was incubated with 1% OsO4 in PB (0.1 M) for 1 h at 4°C. Kidney organoids were fixed in 1% Dehydrated in a graded series of ethanol solutions, and embedded in epoxy resin. Ultrathin sections (70 nm) were cut and stained with uranyl acetate and lead citrate. Slides were then imaged using an G2 Spirit transmission electron microscope (FEI Tecnai, Hillsboro, OR, United States).

## Results

### Generation and characterization of kidney organoids: morphological and molecular features

**FIGURE 1:**
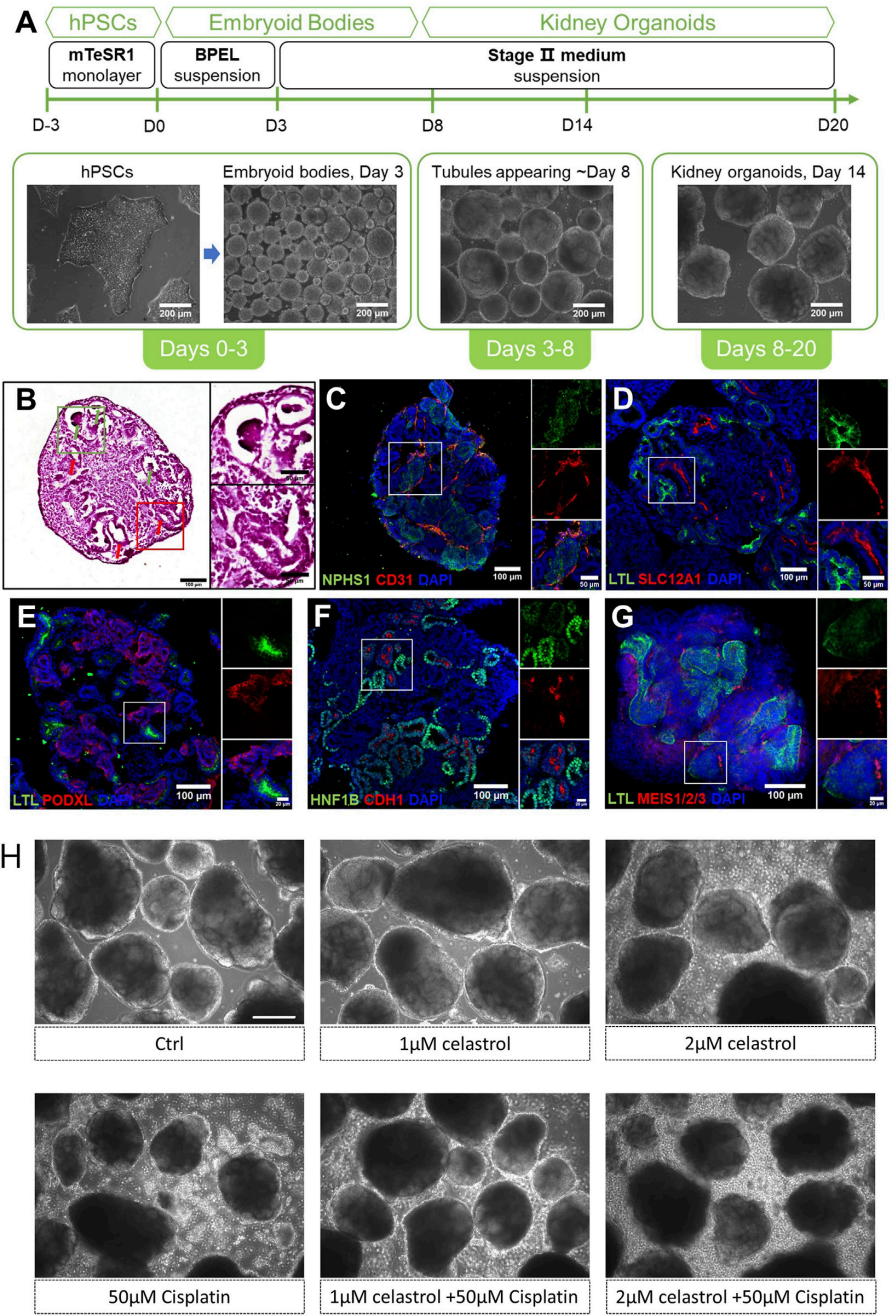
Self-organization and structural profiling of the kidney oragnoids. An overview of our differentiation method for kidney organoids. **(A)** An overview of our differentiation method for kidney organoids and representative images of kidney organoids at different stages. **(B)** Left: H&E staining of kidney organoid sections cultured to day 14, with the red arrows indicating tubular structures and green arrows indicating glomerular structures. Right: a zoom-in view of the red and green boxes. **(C–G)** Immunofluorescent staining of day 14 kidney organoids sections showing NPHS1+ podocytes and CD31^+^ endothelial cells, LTL + proximal tubules and SLC12A1+ thick ascending limb segments, PODXL + podocytes, HNF1B + tubules/collecting duct and CDH1+ distal tubules, and MEIS1/2/3+ interstitial cells. Corresponding zoom-in views of the white boxes are on the right of each image. **(H)** Representative bright field image (10X) of kidney organoids cultured with celastrol or cisplatin or co-treated with both compounds, Bar = 200 μm.

The process of kidney organoids self-organization is illustrated in Figure 1A. At 14 days of culture, the 3D structures presented tubular (red arrows) and glomerular (green arrows) formation (Figure 1B). These constructs expressed NPHS1, a critical protein primarily found in podocytes of the glomerulus, and CD31, a marker highly expressed in endothelial cells, indicating the development and/or maturation of the kidney vasculature (Figure 1C). Additionally, the organoids presented LTL (Green), a highly specific marker of the kidney’s proximal tubules, and SLC12A1 (Red) staining, a marker for identifying the presence of thick ascending limb segments of the kidney (Figure 1D). Organoids contained multiple segmented nephron structures that resemble glomeruli and tubules marked by PODXL + podocytes (Figure 1E), HNF1B + tubules/collecting duct and CDH1+ distal tubules (Figure 1F), and MEIS1/2/3+ interstitial cells (Figure 1G). Typical images demonstrating the morphology of the kidney organoids exposed to celastrol or cisplatin under various conditions are shown in (Figure 1H). After the exposure of the kidney organoids to cisplatin, the organoids structure appeared partly collapsed. In addition, the collapsed structures were significantly reduced at celastrol was added.

**FIGURE 2:**
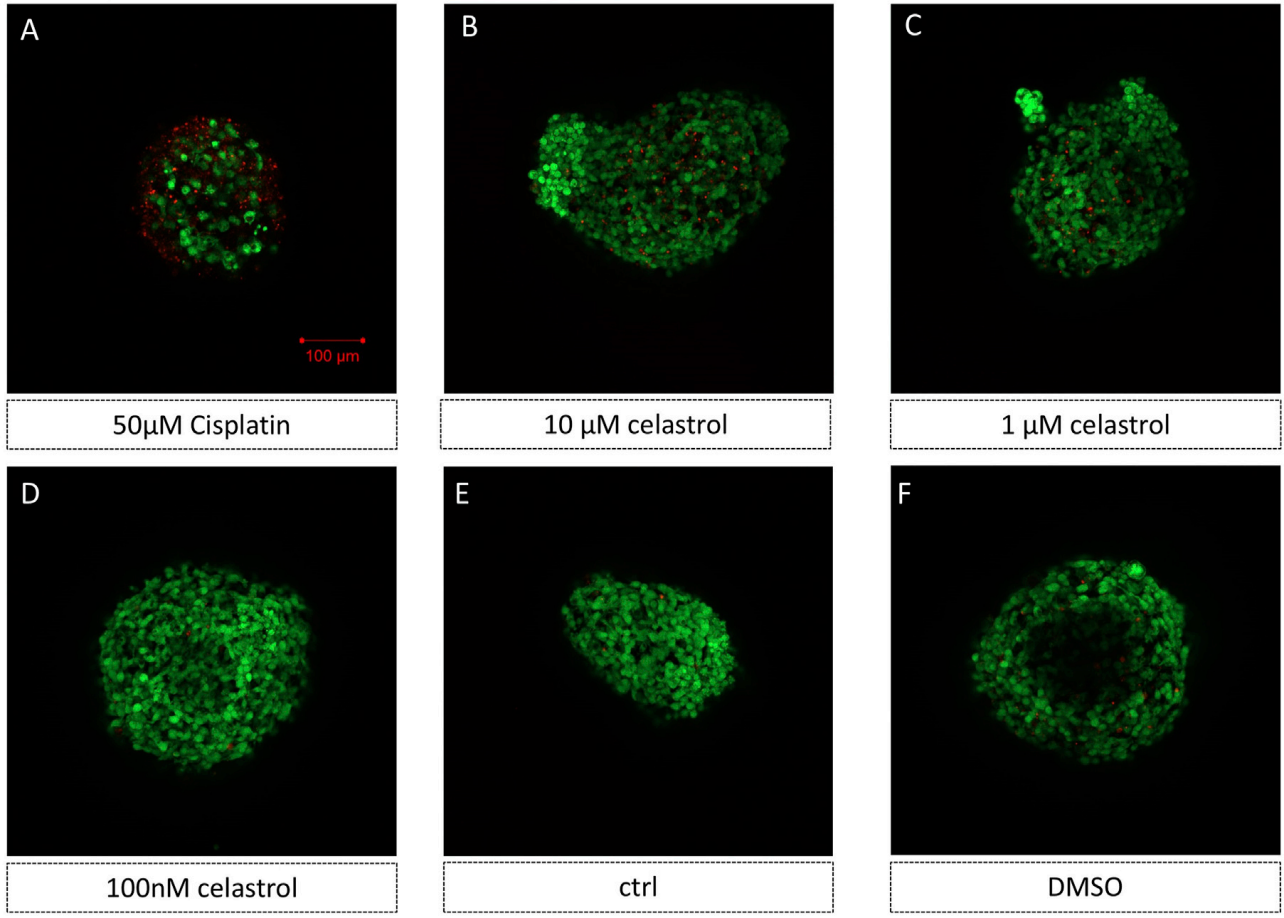
Representative images of 3D kidney oragnoids models showed cisplatin and celastrol induced kidney toxicity (AO/PI staining) n = 5. **(E)** kidney oragnoids not treated with drugs (control), **(A–D, F)** kidney oragnoids treated with: **(A)** 50 µM Cisplatin **(B)** 10 µM celastrol, **(C)** 1 µM celastrol, **(D)** 100 nM celastrol, and **(F)** DMSO.

We used 3D kidney organoid models to investigate the effects of cisplatin and celastrol on drug-induced kidney toxicity. The constructs were exposed to varying concentrations of the compounds to assess their effects on cell viability. Figure 2A shows a significant increase in cell death within organoids treated with 50 µM cisplatin, as evidenced by the increase in PI fluorescence, indicative of membrane damage and cell death. Moreover, the nephrotoxicity of celastrol at 10 µM (Figure 2B), 1 µM (Figure 2C) and 100 nM (Figure 2D) was significantly less pronounced than that observed in constructs treated with cisplatin-50 µM. Furthermore, the number of PI-stained cells in the 100 nM cL group (Figure 2D) was unnoticeable, similar to that found in Control (Figure 2E), and DMSO groups (Figure 2F).

### Kidney organoid models reveal the dose-dependent response relationship of celastrol-induced citotoxicity

**FIGURE 3:**
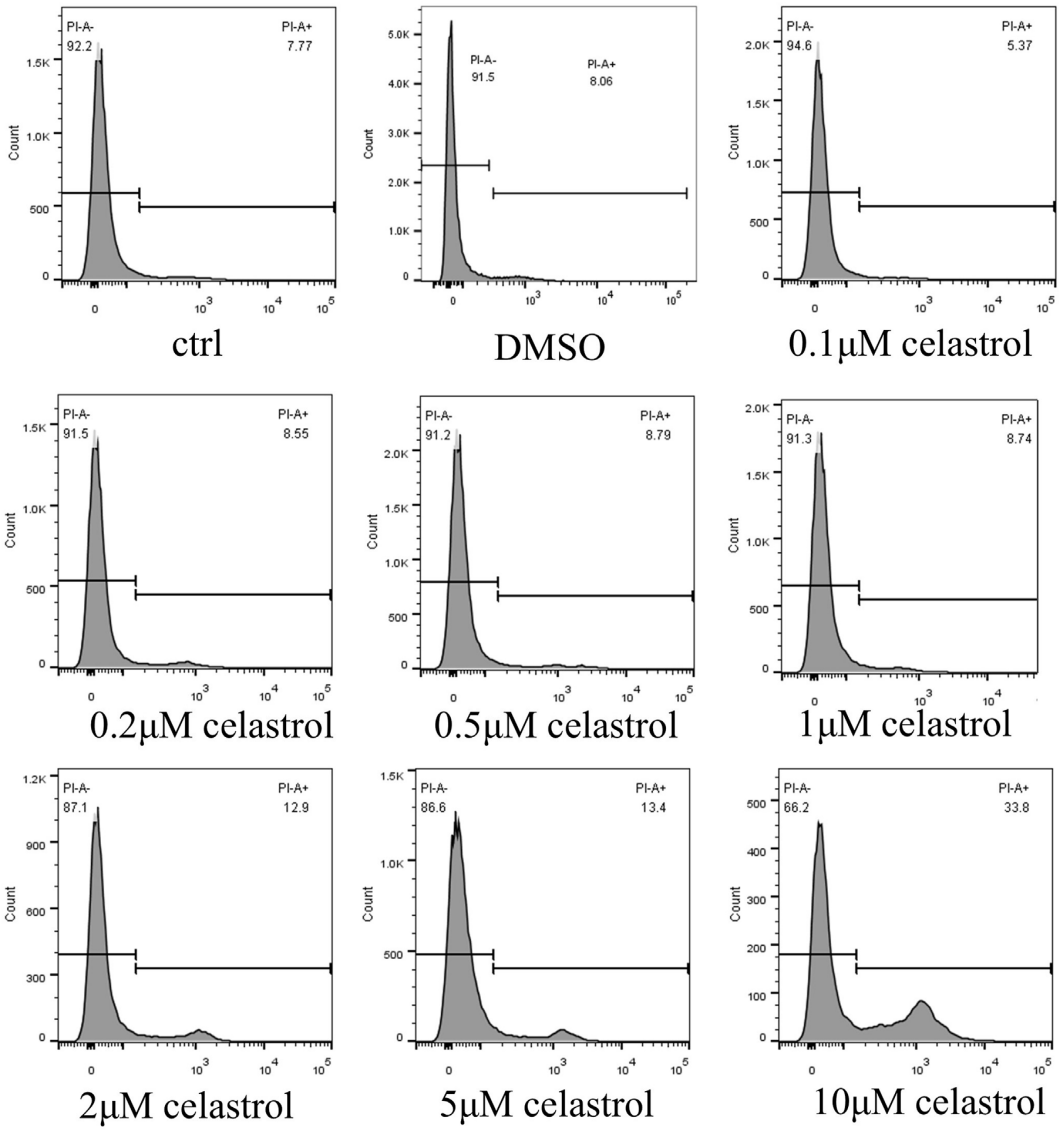
Dose-dependent Kidney Toxicity Induced by Celastrol evaluated by Flow cytometer. The rate of death cell after administration of DMSO or celastrol at 0.1, 0.2, 0.5, 1, 2, 5, or 10 μM for 2 days in kidney organoids. The dose-dependent response of celastrol-induced cytotoxicity was evaluated.

Kidney organoids were exposed to varying concentrations of celastrol (0.1, 0.2, 0.5, 1, 2, 5, and 10 µM) to assess dose-related toxic effects (Figure 3). The 3D constructs were stained with propidium iodide (PI) to determine cell viability. At 10 µM celastrol, the proportion of PI-positive cells reached 33.8%, compared to 7.7% in the control group, while the number of PI-stained cells in the groups treated with Celastrol >1uM remained comparable to that of the basal levels (Ctrl). The Flow cytometer assay findings demonstrated that celastrol induced cytotoxicity in a dose-dependent manner, as evidenced by the increased number of dead cells, with concentrations to produce low cytotoxicity and yet retain the beneficial effects at an optimal range of 1–2 µM.

**FIGURE 4:**
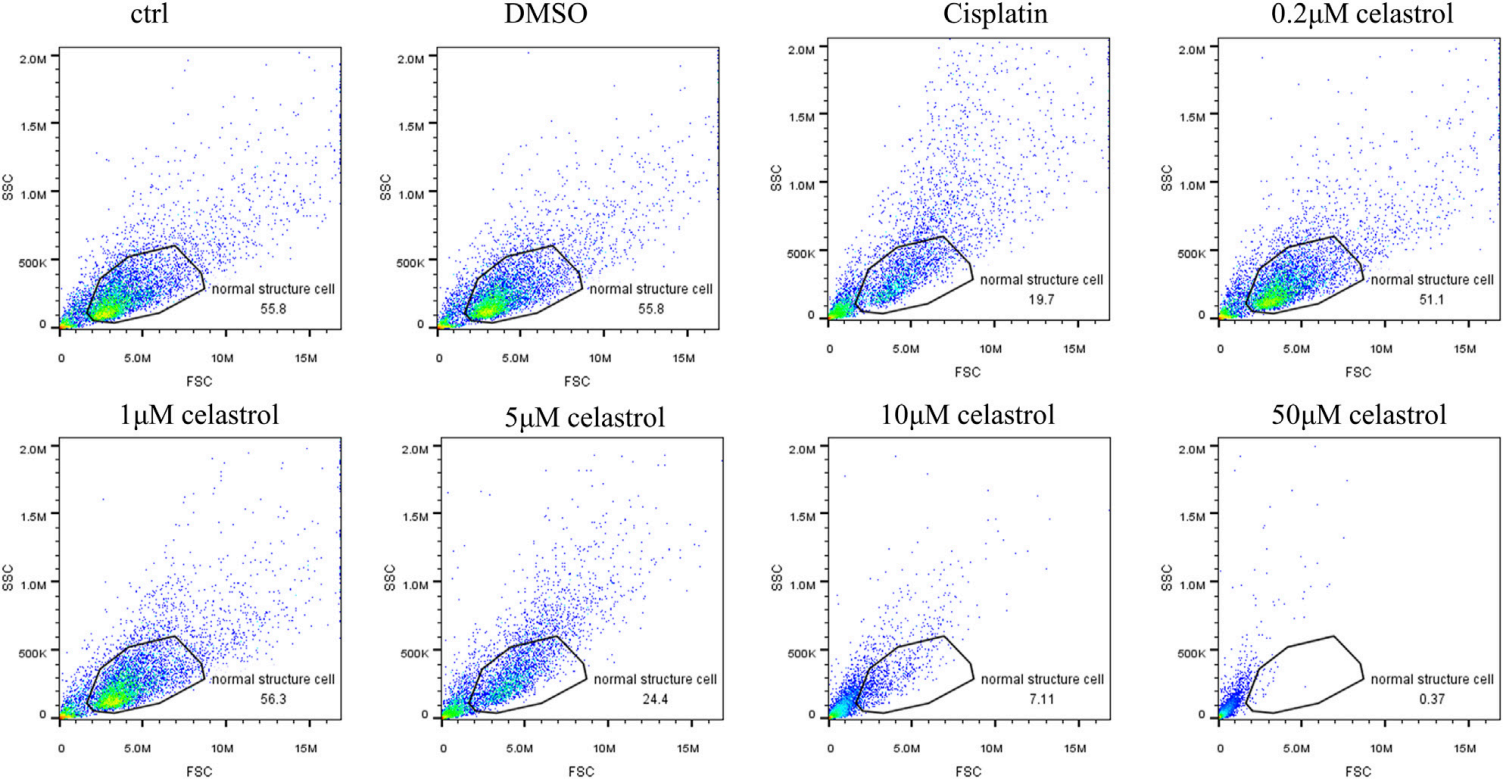
Flow cytometry analysis rate of normal cell structure after administration of celastrol at 0.2, 1, 5, 10μM, or 50 mM for 2 days in kidney organoids. The dose-dependent response of celastrol-induced cytotoxicity was compared with that of cisplatin treatment.

The dose-dependent response of celastrol-induced cytotoxicity was compared with that of cisplatin treatment. Flow cytometry analysis revealed that cisplatin at 50 µM resulted in only 19.7% of cells maintaining a normal structure, in stark contrast to the 55.8% observed in the control (Ctrl) and DMSO groups. Interestingly, Celastrol at 0.2 µM and 1 µM concentrations demonstrated normal structure cell percentages comparable to those in the DMSO and control groups (56.3%). However, at higher concentrations of 5, 10, and 50 μM, celastrol induced a dose-dependent decrease in the percentage of cells with normal structure, underscoring its nephrotoxicity at elevated doses (Figure 4).

**FIGURE 5:**
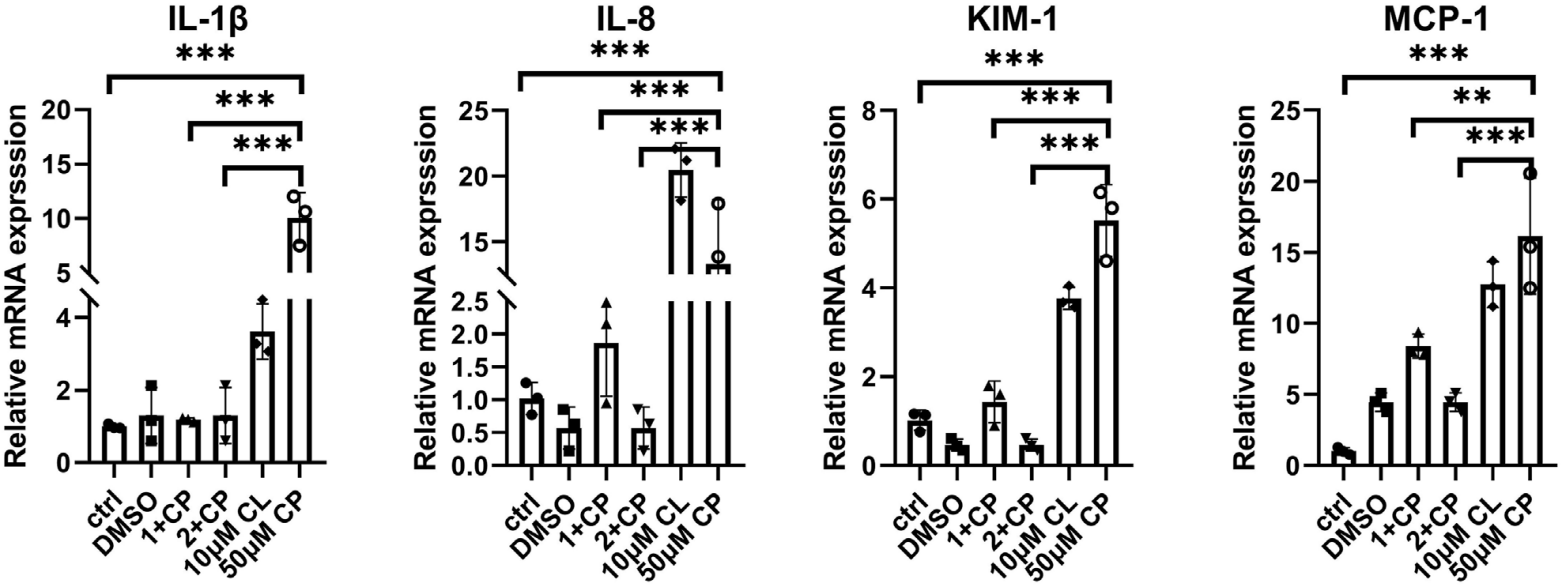
Expression of kidney inflammatory and injury mRNA biomarkers in kidney oragnoids incubated with 1 μM celastrol+50 μM cisplatin, 2 μM celastrol+50 μM cisplatin, 10 μM celastrol, and 50 μM cisplatin for 2 days n = 3. ctrl, control; 1+cp, 1 μM celastrol+50 μM Cisplatin; 2+cp, 2 μM celastrol +50 μM Cisplatin; 10μM CL, 10 μM celastrol; cp, Cisplatin. (n = 3/group, error bars represent the SD, **P ≤ 0.01; ***p ≤ 0.001 in the different groups compared with the cp group using a One-way ANOVA test).

To assess the impact of renal injury induced by individual treatments with cisplatin or celastrol, as well as their combined application, we adopted a holistic measurement strategy that encompasses the simultaneous quantification of several key renal mRNA markers. Specifically, we have focused on the mRNA levels of Interleukin-8 (IL-8), Interleukin-1 beta (IL-1β), Kidney Injury Molecule-1 (KIM-1), and Monocyte Chemoattractant Protein-1 (MCP-1), as these biomarkers are indicative of renal stress, inflammation, and injury (Figure 5). IL-1β and IL-8 are central to the kidney’s inflammatory response, with elevated mRNA levels indicating activation of inflammatory pathways typical of nephrotoxic agents, such as cisplatin. Remarkably, the mRNA levels of IL-1β, and IL-8 in the groups treated with a combination of 50 µM cisplatin and 1 (1+cp), and particularly 2 (2+cp) µM celastrol, were similar to those observed in the control groups (Ctrl, DMSO). Moreover, the mRNA expression levels in the 1+cp and 2+cp groups were significantly lower than those seen in the 10uM Celastrol or Cisplatin 50uM the transcriptional expression of KIM-1, a specific marker for kidney proximal tubule injury, showed similar expression patterns and were downregulated to basal levels when organoids were subjected to 50 µM cisplatin combined with 1 and 2 µM celastrol. However, MCP-1, known to recruit monocytes and macrophages to inflammation sites that can exacerbate tissue damage, also presented similar expression patterns to those observed in the previously described biomarkers. However, 1+cp did not bring MCP-1 mRNA levels to control levels. Taken together, by measuring these biomarkers, we can gain insights into the mechanisms of celastrol-induced nephrotoxicity, predict the extent of kidney damage, and potentially guide dosage adjustments to mitigate adverse effects, thus providing a holistic view of the nephrotoxic potential of the compound.

### Celastrol alleviated renal glomerulus and proximal tubules injury

**FIGURE 6:**
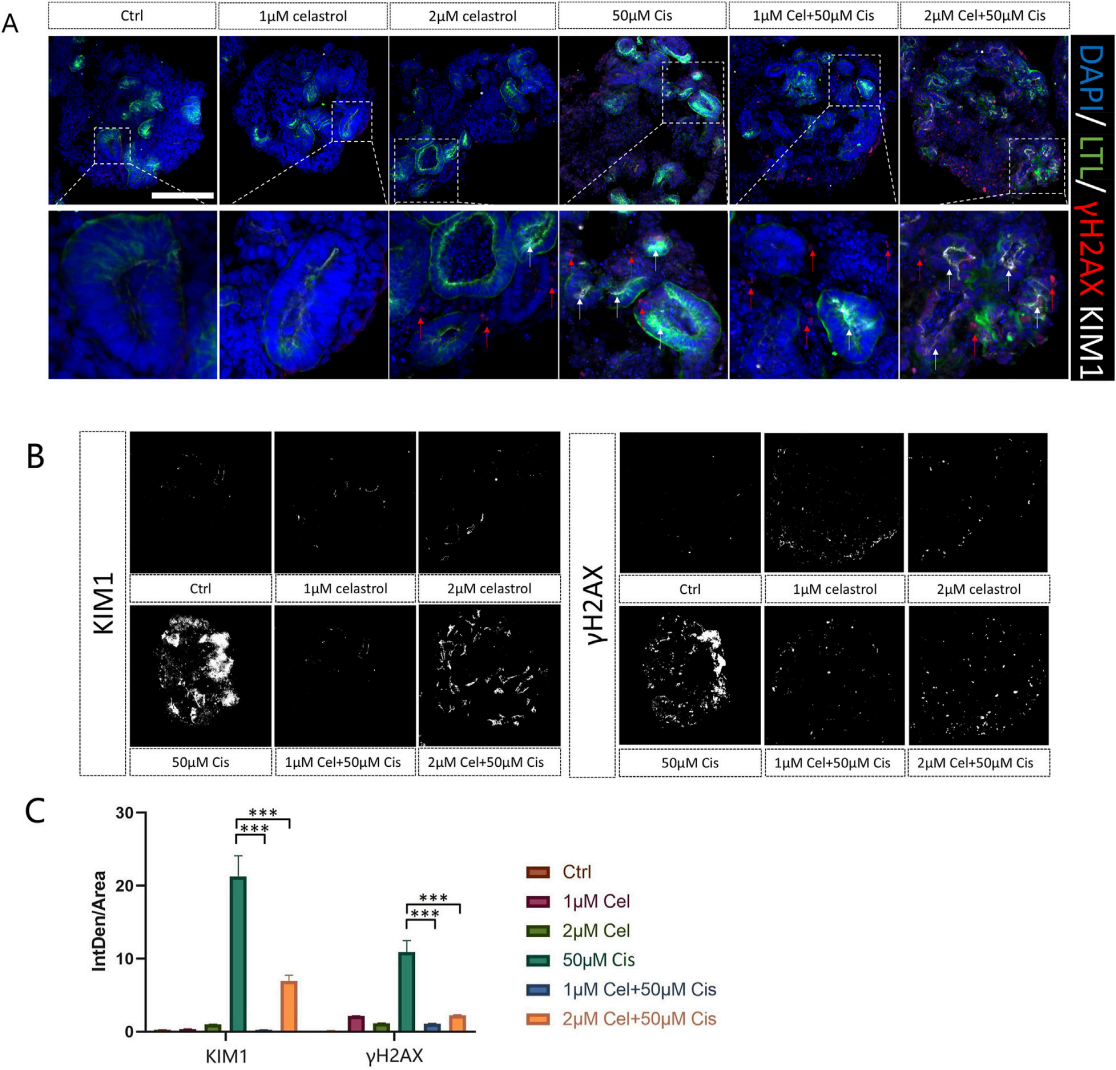
**(A)** Representative images of kidney injury (γH2AX, KIM1) in LTL of kidney organoids after administration of celastrol at 1 and 2 μM, or Cisplatin 50 μM for 2 days n = 3. Bar = 100 μm. **(B)** Quantification of γH2AX and KIM1 fluorescence intensity. n = 5. **(C)** The fluorescence intensity vs. area. Ctrl, control; 1 μM Cel, 1 μM Celastrol; 2 μM Cel, 2 μM Celastrol; 50 μM Ci, Cisplatin; 1 μM Cel+50μMCis,1 μM celastrol+50 μM Cisplatin; 2 μM Cel+50μMCis, 2 μM celastrol +50 μM Cisplatin; (n = 5/group, error bars represent the SD, ***p ≤ 0.001 in the different groups compared with the 50 μM Cisplatin group using a Kriskall-Wallis test).

Figure 6A shows representative images of kidney injury markers (KIM-1 is a specific and sensitive biomarker of proximal tubules of kidney injury, γH2AX indicates DNA damage) in LTL-stained kidney organoids following the administration of celastrol at concentrations of 1 μM and 2μM, cisplatin at 50μM, or a combination of both drugs, for a duration of 2 days. The fluorescence intensity was showed in (Figure 6B), determined using the ImageJ software, revealed the cytotoxic effects of cisplatin at 50μM, as indicated by the elevated expression of both renal damage markers (Supplementary Figure 1). Notably, the co-administration of cisplatin 50 μM with 1μM and 2 μM celastrol not only mitigated drug-induced kidney injury, but also demonstrated protective actions. These findings suggest that 1 and 2 µM celastrol could potentially offer a novel therapeutic strategy that balances the potent anticancer action of cisplatin with the cytoprotective benefits of celastrol, thereby enhancing the overall safety and efficacy of cancer treatment.

**FIGURE 7:**
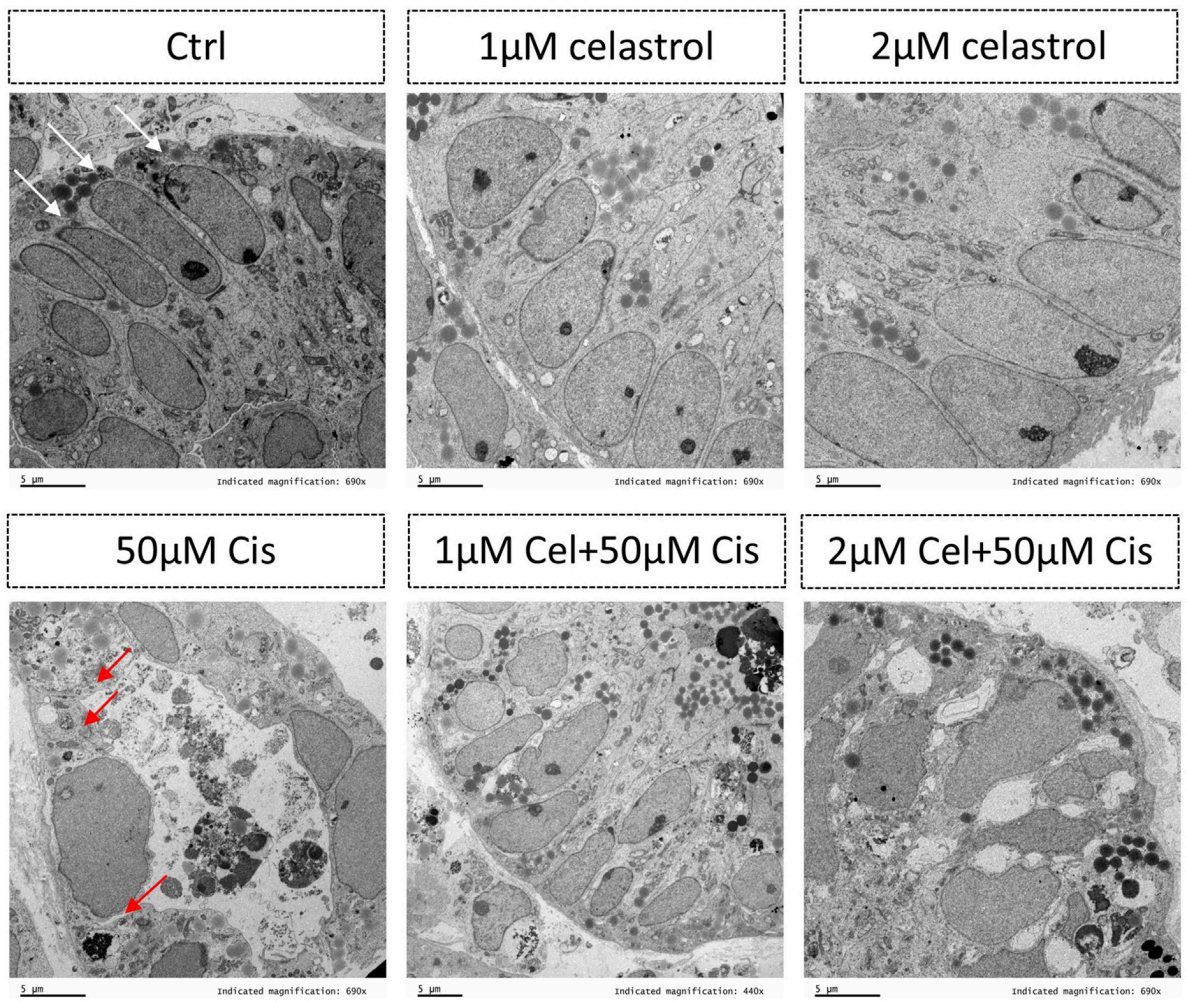
Transmission electron microscopy showing the presence of tubular cells within the kidney organoids. The white arrows indicate normal tubular cells while red arrows indicating damaged tubular cells after injury. Ctrl, control; Cel, celastrol; Cis, cisplatin.

In this study, human pluripotent stem cells derived kidney organoids comprised of not only complex interacting component cell types, but also some distinct segmenting nephrons, including distal tubule, proximal tubule, foot processes and podocytes of the glomerulus (Figure 1). With such various of kidney cell types, the advantages for use of these nephrons for nephrotoxicity screening have significantly promoted (Figure 7). Cisplatin induced-kidney injury was characterized by TEM, in cisplatin group, clear damages in tubular cells were identified, such as loss of cells shape, necrosis of renal tubular epithelium and vacuoles formation. 1 μM celastrol + cisplatin group resulted the significant injury recovery in tubular cells comparing with others.

### Downregulation of SNORD3A and mir3615 by celastrol mitigates cisplatin-induced nephrotoxicity in kidney organoids

**FIGURE 8:**
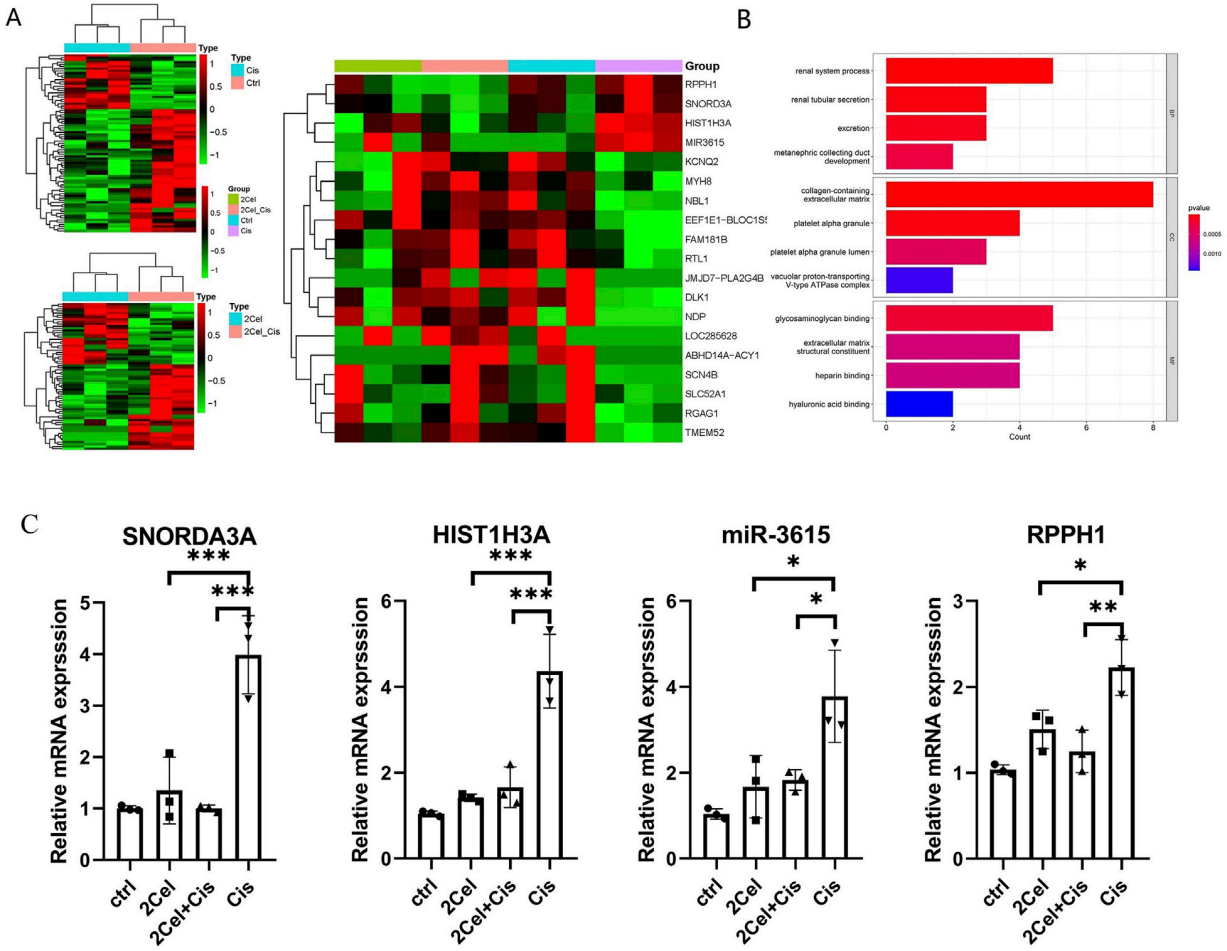
**(A)** Heatmap of up-and downregulated DEGs of different groups (Cisplatin vs. Control, 2 μM Cel + Cis vs. Cis, Four groups compare). **(B)** The GO barplot of 2 μM celastrol + Cisplatin vs. Cisplatin group. The diagrams showing the most differentially expressed functional pathways regarding the renal system, renal tubular secretion and excretion based on the GO database. **(C)** Validation of mRNA and miRNA by RT-qPCR. Relative expression level of HIST1H3A and specific ncRNAs SNORD3A, miR-3615, and RPPH1 in kidney organoids treated with 2 μM celastrol, 50 μM Cisplatin or 2 μM celastrol+50 μM Cisplatin for 2 days. Relative quantifcation was determined by normalization to GAPDH or U6. (n = 3/group, error bars represent the SD, *P ≤ 0.05; **P ≤ 0.01; ***p ≤ 0.001 in the different groups compared with the cp group using a One-way ANOVA test).

For the RNA-seq result, compared 2 μM celastrol + cisplatin vs. Cisplatin group, GO analysis shown the most differentially expressed functional pathways are the renal system, renal tubular secretion and excretion function based on the GO database, indicating celastrol regulate renal tubular secretion and excretion function with protective effect on glomerulus. Meanwhile, Figure 8 shows the significant upregulation of SNORD3A in kidney organoids treated with cisplatin (Cis), with high expression levels correlating with nephrotoxicity. SNORD3A was primarily enriched in tubular epithelial cells in response to acute kidney injury (AKI) in tubular epithelial cells. We also observed a dramatic decline in SNORD3A expression in the celastrol + cisplatin group (2Cel-Cis) (Figure 8C), indicating that celastrol played a pivotal role in the inhibition of cisplatin-induced nephrotoxicity. Previous studies have demonstrated that miRNAs are involved in the pathophysiology of AKI, and aberrant miRNA expression levels serve as biomarkers for diagnosing AKI miRNA signatures. Our RNA-seq and RT-qPCR results showed that miR-3615 was positively associated with cisplatin-induced kidney injury. Moreover, miR-3615 was significantly downregulated in the 2Cel-Cis, further indicating its positive relationship with cisplatin-induced nephrotoxicity (Figure 8C). Furthermore, RPPH1, which is upregulated in diabetic nephropathy via an interaction with Gal-3, and the HIST1H3A gene, both presented expression patterns consistent with what we’ve observed for the previous analyzed genes, also indicating a possible relationship with the protective actions of Cis/Cel cotreatment (Figure 8C).

## Discussion

Here, we present a comprehensive evaluation of kidney organoids self-organization using our refined differentiation method. This evaluation shows their structural and molecular characteristics, which closely mimic those of the native kidney tissues. The constructs displayed markers indicative of renal development, such as NPHS1+ podocytes and CD31^+^ endothelial cells, which are crucial for glomerular and vascular formation (Figure 1D). Additionally, SLC12A1+ and LTL + staining confirmed the differentiation of thick ascending limb segments and proximal tubules (Figure 1E), which are essential components of the renal filtration system. At the same time, there are a variety of techniques that were utilized to the measurement of function and structure of kidney organoids, including immunofluorescence, RNA-seq, Flow cytometer, and TEM.

The kidney organoid model was successfully utilized to assess the nephrotoxic effects of cisplatin and cisplatin-induced nephrotoxicity reversal by cotreatment with celastrol. Consistent with the literature (Tang et al., 2023; Belmonte-Fernández et al., 2023), in our study, we observed a significant increase in cell death in organoids treated with 50 µM cisplatin (Figure 2A) compared to those in the celastrol-treated and control (ctrl, DMSO) groups. Celastrol’s dose-dependent nephrotoxicity (Figure 3) is a novel observation that adds to the existing body of knowledge on the compound’s biological effects. Lower concentrations of celastrol (1–2 µM) showed minimal toxicity, suggesting a potential therapeutic window where celastrol could exert its anti-inflammatory and anti-neoplastic effects without causing significant kidney injury. The comparative analysis of cytotoxic effects between cisplatin and celastrol treatments (Figure 4) revealed that celastrol, at certain concentrations, could mitigate cisplatin-induced nephrotoxicity. This is a significant finding, as it suggests that celastrol may have a protective role against kidney injury, possibly through its antioxidant and anti-inflammatory properties (Allison et al., 2001; Yang et al., 2006). Though, potential toxicity restricts its further application. Immortalized cell models like human proximal tubule epithelial cell line (HK-2) and mouse renal tubule epithelial cells (RTECs) are commonly used for nephrotoxicity assessment. However, absence of essential molecular structures and cellular components makes its data not easily translated to equivalent values *in vivo*. Significant differences in celastrol safety values were found between cell lines and kidney organoids. It was reported that the highest tolerant values of celastrol on HK-2 and RTECs were 50 nM ((Yu et al., 2018)). The dose we used in kidney organoids in the present study was much more than above values. The celastrol concentrations to generate low nephrotoxicity and yet remain the beneficial effects at an range from 1 to 2 µM.

The combination of cisplatin and celastrol resulted in downregulation of inflammatory and injury biomarkers (Figure 5), indicating a potential synergistic effect. Normalization of IL-1β, IL-8, KIM-1, and MCP-1 mRNA levels in co-treated organoids suggests that celastrol may modulate the inflammatory response and promote tissue repair, a promising avenue for future research. Moreover, fluorescence intensity analysis of γH2AX and KIM-1 has shed light on the potential benefits of co-administering cisplatin (50 µM) with celastrol (1–2 µM) in mitigating drug-induced kidney injury. This co-administration may contribute to the complex mechanisms by which celastrol provides cytoprotection. Phosphorylation of the Ser-139 residue on the histone variant H2AX, resulting in the formation of γH2AX, represents an early cellular response to DNA double-strand breaks. Detection of this phosphorylation event is recognized as a highly specific and sensitive molecular marker for monitoring the onset and resolution of DNA damage. Importantly, this marker has been linked to oxidative stress and the production of reactive oxygen species (ROS) (Mah et al., 2010). Therefore, upregulation of γH2AX in response to cisplatin may serve as an indicator of oxidative stress and ROS production during drug-induced kidney injury (DIKI).

Additionally, we examined the expression of several transcription factors. Among them, HIST1H3A and the non-coding RNAs (ncRNAs) SNORDA3A, miR-3615, and RPPH1 showed similar expression patterns, which were positively correlated with the cytotoxicity features of 50 µM cisplatin and the protective action of 2 µM celastrol. HIST1H3A is one of the genes that encode the histone H3.1 protein, and histone modifications have been implicated in both the development and progression of kidney diseases as well as AKI (Kato and Natarajan, 2019; Wang et al., 2022; Pan et al., 2024). For instance, phosphorylation of histone H3 on serine residue 10 (H3Ser10) has been linked to endothelial activation in diabetic kidney disease, facilitating the recruitment of inflammatory cells that contribute to kidney injury and fibrosis (Alghamdi et al., 2018). On the other hand, Zhu et al. (2024) observed that deficiencies in SNORDA3A exhibit a mitigating effect on the stimulator of interferon gene (STING)-associated ferroptosis phenotypes and the progression of kidney tubular injury. Mechanistically, SNORDA3A regulates the STING signaling axis by promoting STING gene transcription, and the administration of SNORDA3A antisense oligonucleotides represents a significant therapeutic advantage in a mouse model of AKI (Zhu et al., 2024). Consistent with Zhu’s study, we observed a significant upregulation of SNORD3A in response to cisplatin-induced nephrotoxicity and its subsequent downregulation upon celastrol cotreatment, suggesting that the protective effects of celastrol at 1 or 2 µM might be partially related to the inhibition of the progression of tubular injury (Figure 8). Although the link between miR-3615 and kidney injury has not been well documented in the available scientific literature, we analyzed its transcriptional expression. These results indicated that miR-3615 was positively associated with cisplatin-induced kidney injury and celastrol-induced cytoprotective action. Furthermore, RPPH1 (ribonuclease P RNA component H1), a critical component of the ribonuclease P complex involved in the maturation of tRNA molecules by cleaving their 5′leader sequences, showed expression patterns similar to those of the other ncRNAs. RPPH1 plays a fundamental role in cellular RNA processing and is essential for proper functioning of the ribonuclease P enzyme complex. However, in the context of AKI and DIKI, particularly cisplatin-induced nephrotoxicity, the specific mechanisms by which RPPH1 might be involved have not yet been elucidated.

Taken together, the potential therapeutic advantages of targeting HIST1H3A and specific ncRNAs such as SNORD3A, miR-3615, and RPPH1 further support the complex interplay between these genomic elements and drug-induced nephrotoxicity. With the rise of organoid technologies, it has become possible to studying various diseases that affect the kidneys as well as a preclinical model for drug toxicity screening and to investigate the structure and molecular changes occurring in a more physiologically relevant environment.

## Conclusion

Our kidney organoid model has been proven to be an invaluable tool for advancing our understanding of nephrotoxicity. Its robustness lies in its ability to closely mimic the structural and functional characteristics of native kidney tissues, providing a reliable and controllable environment for studying the mechanisms of DIKI, such as those induced by cisplatin.

Moreover, our findings underscore the significance of a cotreatment approach using cisplatin and celastrol. at concentrations of 1 and 2 μM, celastrol has been demonstrated to complement the full anti-neoplastic potential of cisplatin in cancer treatment with its own cytoprotective actions. This synergistic combination offers a promising avenue for enhancing the therapeutic efficacy of cisplatin, which is a widely used chemotherapeutic drug. By reducing its cytotoxic side effects on the kidneys while maintaining its anticancer potency, it is possible to envision a new paradigm in cancer therapy that is both effective against tumours and protects normal tissues. However, the therapeutic window of celastrol is very narrow (from 1 to 2 µM), associated with the occurrence of side effects. Further research is warranted to fully realize the clinical potential of these findings and studies on human clinical trial are required.

## Data Availability

The raw data presented in the study are deposited in the National Genomics Data Center, accession number OMIX007089; available at https://ngdc.cncb.ac.cn/omix/releaseList.
