# Supplementary figures and images for: Celastrol reduces cisplatin-induced nephrotoxicity by downregulating SNORD3A level in kidney organoids derived from human iPSCs
Source: Front Pharmacol. 2025 Mar 27;16:1464525. doi: 10.3389/fphar.2025.1464525 (PMC11983408; doi:10.3389/fphar.2025.1464525)

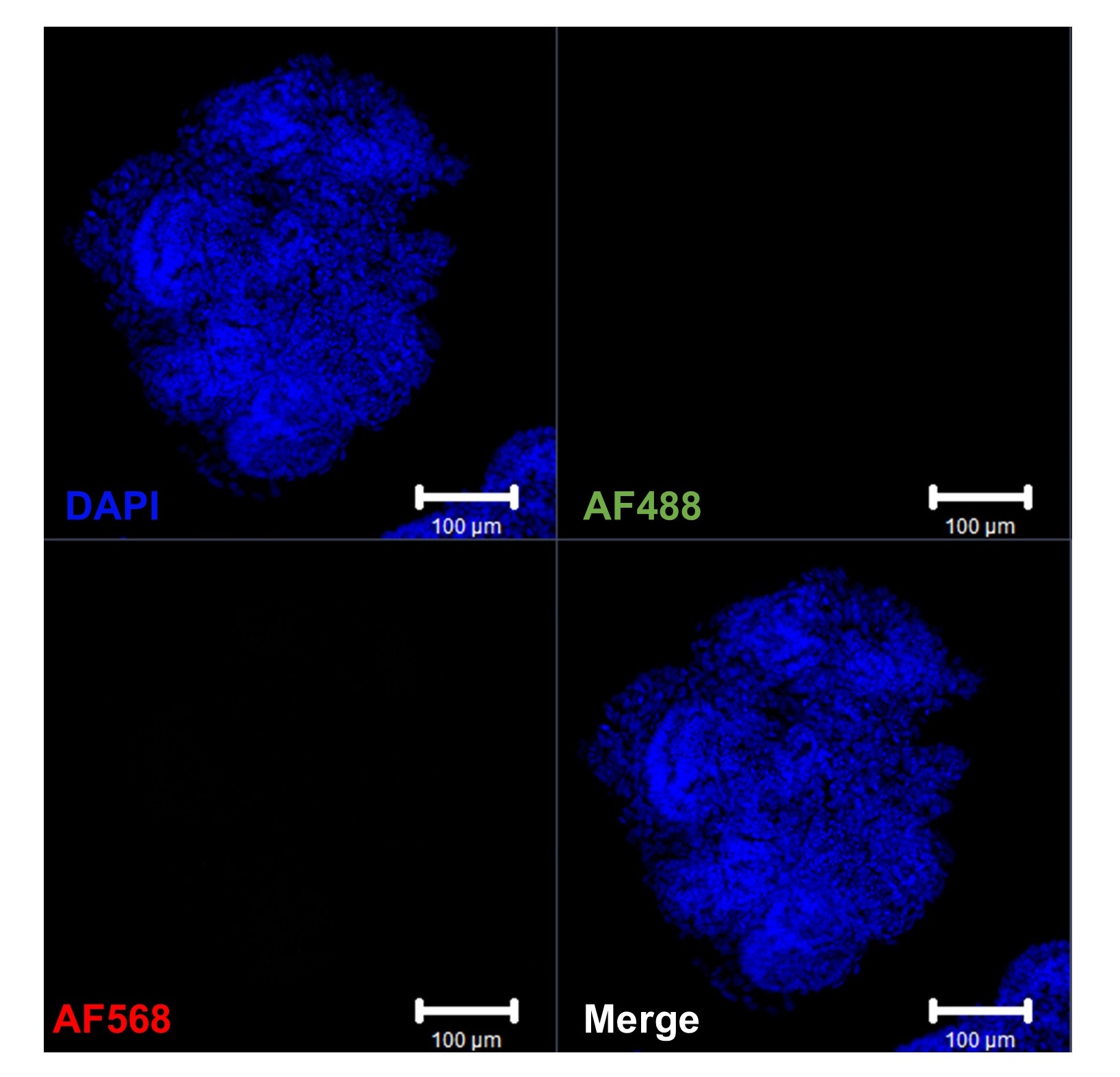

Supplement: Supplementary file 1 [file Image1.jpeg]
